# The impact of cash transfer participation on unhealthy consumption in Brazil

**DOI:** 10.1016/j.hpopen.2022.100087

**Published:** 2022-12-06

**Authors:** Fernanda Araujo Maciel, Diogo Duarte

**Affiliations:** a California State University, Sacramento, College of Business, 6000 J Street, Sacramento, CA 95819, USA; b Florida International University, College of Business, 11200 S.W. 8th St., 236, Miami, FL 33199, USA

**Keywords:** Conditional cash transfer program, Unhealthy consumption, Propensity score matching estimation

## Abstract

We assess the impact of the Brazilian government’s conditional cash transfer program Bolsa Família on unhealthy consumption by households, proxied by expenses with ultra-processed food, alcohol, and tobacco products. Using machine learning techniques to improve the propensity score estimation, we analyze the intensive and extensive margin effects of participating in the program on the household purchase of unhealthy products. Our results reveal that program participants spend more on food in general, but not necessarily more on unhealthy options. While we find evidence that participants increase their probability of spending more on food away from home, they do not significantly alter their expenditures on packaged food, alcohol, or tobacco products.

## Highlights

• Brazilian cash transfer participants of the Bolsa Familia program spend more on food.
• Recipients do not spend more on unhealthy products, such as packaged foods.
• There is no increase in alcohol or tobacco products purchased.
• Machine learning algorithms improve the propensity score estimation of the model.

## 1 Introduction

Conditional cash transfer (CCT hereafter) programs aim to alleviate poverty and improve the nutrition of low-income families, among other goals. The literature has shown that families participating in these programs use the funds to purchase more food, which ultimately improves their caloric consumption [1], [2]. However, the nutritional quality of the food purchased is still low. For instance, in Mexico, participants in the *Oportunidades* program improve their dietary intake of iron, zinc, and vitamin A, but this effect is due to the supplementary nourishment provided by the government and not due to an improvement of their diet [3]. In the United States, Supplemental Nutrition Assistance Program (SNAP hereafter) participants consume as many calories as nonparticipants. However, SNAP participants have systematically partaken in lower dietary quality than nonparticipants [4]. This suggests that SNAP participants fulfill their caloric needs by purchasing high-energy-dense foods that are poor in nutrients.

Our study further explores the implications of conditional cash transfer programs for the diet of their participants. Using the database on the Brazilian conditional cash transfer program, called *Bolsa Família*, we examine (1) whether participants in the program increase their food expenses overall and (2) if the increase in food expenses among these participants translates into a higher expenditure in high-caloric, low-nutritious (i.e. ultra-processed) food. Our results show that while program participants purchase more food overall, the share of ultra-processed food does not increase with the additional funds provided by the government. This result has important implications for policy design since the overconsumption of fat and sugar may lead to the development of diseases such as diabetes, hypertension, allergies, and cancer [5], [6], [7].

A second important finding is that while participants in *Bolsa Família* have a higher probability of purchasing food away from home and cookies, the probability of participants purchasing soda decreases. Interestingly, we find that the program participants do not significantly change their expenditures on packaged food, alcohol, or tobacco products (which include cigarettes, hand-rolled cigarettes, cigars, pipes, and cigarillos).

Our study also contributes to the methodological front. To evaluate the impact of the conditional cash transfer program on the unhealthy consumption of these families, we use propensity score matching (PSM hereafter). PSM estimation relies (mostly) on logistic regression [8], which requires the model to be parametric and linear (in terms of the log of the odds). As a result, the estimates produced by this method can be severely biased in the presence of mild nonlinear relationships. To overcome this problem, we follow recent studies in the literature that suggest that machine learning techniques can enhance the predictability of econometric models and reduce estimation biases [9], [10], [11], and compare the propensity score from a standard logistic regression model with the ones obtained from four popular machine learning models: random forests, gradient boosting, support vector machines (SVM hereafter), and neural networks. Our analysis suggests that the propensity score from the gradient boosting method has the highest sensitivity and the lowest misclassification. After computing the propensity score of the gradient boosting method, we proceed to the second stage of the PSM, which consists of matching the propensity scores of families that participate in the program and those who do not, to unveil the effects of participating in the *Bolsa Família* program. To the best of our knowledge, this is the first study to provide a comprehensive analysis of the performance of these machine learning algorithms on the estimation of propensity scores with this dataset.

The remainder of this paper is organized as follows. Section 2 contains a brief description of the Brazilian cash transfer program *Bolsa Família* and its relationship with ultra-processed foods. Section 3 contains our empirical analysis, with the description of the dataset, methodology, and results. Section 4 concludes.

## 2 *Bolsa Família*: The Brazilian CCT Program

The central concept of any CCT program is to condition the received funds provided by the government to households in poverty on the participants’ compliance with some health and education requirements [12]. Implemented in 2003, the Brazilian CCT program *Bolsa Família* is the largest social welfare program in the country, covering a quarter of all Brazilian households. The program was introduced with the objective of reducing hunger, malnutrition, poverty, and familial deprivation, while providing low-income families access to public services, such as health, education, and social assistance.

Only families living in extreme poverty are eligible to participate in this program, those with monthly income below R$89 per capita (approximately US$23 per capita, using the conversion rate from 2009, as all values hereafter). On top of the base value of R$89, families receive an additional value of R$41 for each child and pregnant woman or nursing mother (up to five). Families with monthly income below R$178 per capita (approximately US$45 per capita) are also eligible if they have children under 16 years old, or if they are pregnant women or nursing mothers. However, for these families, the benefits are restricted to R$41 per child, pregnant women, or nursing mothers.

Once eligible, families have to comply with the requirements for health and schooling. In addition, the household is enlisted in a national registry, namely *Cadastro* Ú*nico* that contains self-reported information on household demographic characteristics, income, and prior participation in transfer programs. Although all households are free to register in *Cadastro* Ú*nico*, each municipality has quotas allocated by the federal government according to a poverty assessment based on poverty maps [13]. Since officials of each municipality are responsible for the registration process, there is substantial heterogeneity across municipalities in targeting registration [14].

*Bolsa Família* has a substantial impact on the income of the families participating in the program. For example, a family of two parents with three children that has a total monthly income of R$400 (i.e., a monthly income below R$89 per capita) can receive a benefit up to R$212, which corresponds to an increase of more than 50% of their original total monthly income.

One of the main effects of this CCT program is that participants in *Bolsa Família* use the additional funds to increase their standards of living by raising their food consumption. About 87% of the participant families use the cash transfer primarily to purchase food [15]. Beneficiaries spend, on average, 56% of their household income on food. In a study with 5,000 households selected from the *Bolsa Família* registry, the authors show that families report significantly higher consumption of cereals, processed foods, meat, milk and other dairy products, beans, and sugar [16].

While the aforementioned studies have shown an increase in food consumption and a reduction in the food insecurity of these families, it is still unclear whether the increase in calories is associated with good nutritional practices or it is mainly caused by an increase in the consumption of ultra-processed foods. This question is central to policymakers since the increase in the consumption of ultra-processed food is accompanied by diseases, such as diabetes, hypertension, allergies, and cancer [5], [6], [7]. For this reason, we investigate in this study if the increase in food consumption experienced by *Bolsa Família* participants is accompanied by an increase in their consumption of ultra-processed foods.

### 2.1 Ultra-processed foods

Ultra-processed foods (UPF hereafter) are obtained by processing food ingredients such as hydrogenated oils, hydrolyzed proteins, and starch-modified sugars. The ingredients in ultra-processed products are additives to make the final product convenient, cheap, and flavorful [17]. As a result, the products contain a high amount of calories, fat, sugar, and/or salt. Typical examples of UPF are bread, chips, soft drinks, and processed meat [18].

UPF are predominant in high-income countries, and their popularity is fast increasing in middle-income countries as well [19]. Ultra-processed foods are particularly attractive to households with children and to those of lower income and lower education [6]. In the United States, households participating in the SNAP program have higher spending on UPF and lower spending on healthy foods, such as fruits and vegetables, compared to purchases made by nonparticipants in SNAP [20]. A similar pattern is observed in Mexico, where households participating in the CCT program *Oportunidades* are associated with a higher body mass index (BMI hereafter) and a higher prevalence of excess weight and obesity [21].

The same consumption trend of ultra-processed foods can be found in Brazil [18]. Using data from three household budget surveys across three decades (1987, 1995, and 2003), the authors show that the consumption of ultra-processed food products increased among both lower and upper income groups in Brazil. The most recent survey reveals that ultra-processed food products represent 28% of the total energy (418 kcal per capita) a Brazilian household purchases. The survey also documents an increase in sugar, saturated fat, and sodium over the past decades, while calories consumed from *in natura* or minimally processed foods decreased over the past years [22]. A study using the Brazilian Household Budget Survey data from 2009 finds that the availability of ultra-processed products to a household is positively associated with the average household BMI and the prevalence of obesity [23].

The increasing consumption of UPF in past decades motivates us to investigate if this trend is also observed among *Bolsa Família* participants. The next section presents the dataset, methodology, and results from our analysis.

## 3 Empirical analysis

### 3.1 Data

We use data from the Brazilian Household Budget Survey (*Pesquisa de Orçamentos Familiares*, POF hereafter), conducted by the Brazilian Institute of Geography and Statistics (*Instituto Brasileiro de Geografia e Estatística*, IBGE hereafter). The survey contains information about 55,970 households, totaling 190,159 individuals, collected from 2008 to 2009.

The main objective of the POF survey is to investigate patterns of consumption and expenditure of the Brazilian population. This data serves as input for the construction of consumption baskets used to estimate IBGE’s consumer price indexes, such as the IPCA, the main consumer price index in Brazil. POF contains information on individuals (age, level of education, and income), housing (size, the existence of sewage, and type of walls), expenditure for each household (habitation, clothing, health, and food), and source of income (total income, income from social programs including *Bolsa Família*).

For this article, we exclude heads of households that are under 18 years of age. We also exclude families with total monthly income below R$1 and those participants who claim to have a monthly wage *per capita* of above R$6,000 (approximately US$3,000). We perform a pre-trimming of the data and select households that receive up to R$465 (US$233), the monthly minimum wage in 2009. The final sample comprises 33,395 households. Out of the 33,395 households, 8,829 of them (approximately 26.4%) participate in *Bolsa Família*. The average monthly income of the 33,395 households (i.e., recipients and nonrecipients of *Bolsa Família*) is R$870.19, while the average monthly income of the 8,829 *Bolsa Família* recipients without government assistance is R$609.22. These families that participate in the CCT program receive, on average, R$86.32 per month from the program, which corresponds to an increase of 14% of their average income.

Table 1 presents some descriptive statistics on the demographics of the sample. The average age of the household reference person is 46 years old and 31% is female. The most prevalent race designation is multiracial (51%), followed by white (37%), and black (11%). Approximately 70% of the household reference persons have eight years or less of schooling. The average family size is 3.8 people, with 2.04 children among households that report a nonzero number of children. The average household has a monthly per capita income of R$232. The study sample households are predominantly located in the Northeast (37%), followed by the Southeast (35%), with 77% of them living in urban areas. The percentage of families under the poverty line is 24%, with 11% of them living in a situation of extreme poverty.

**Table 1:** Descriptive statistics of the study sample.

**Household Reference Person**		**Household**	
Age	46	Family size	3.75
Female	31%	Number of children	2.04
**Race**	(%)	Household income	R$232.05
White	37	Extreme Poverty	11%
Black	11	Poverty	24%
Asian	0	Pregnant	4%
Multiracial	51	Breastfeeding	8%
Indigenous	1	Urban area	77%
Undeclared	0		
**Schooling**	(%)	**Region**	(%)
⩽4 years	38	North	9
4–7 years	32	Northeast	37
8–10 years	13	Southeast	35
11–14 years	15	South	12
15 + years	2	Center	7

The variables of investigation are the amount spent (in Brazilian Reais) on unhealthy products. More specifically, we focus on household expenses for soda, cookies, packaged foods, food away from home, alcohol, and tobacco products in the month before the survey was conducted. The variable food away from home is divided into two groups: *unhealthy* and *total*. The former includes pastries and snacks, and the latter consists of all food purchased away from home, including unhealthy options. We select the variables *household food expense* and *total food expense*, which also includes food consumed away from home, to estimate the increase in food expenditure among the program participants.

Table 2 shows the percentage of households with nonzero expenditures in our variables of interest and the average household monthly expenditure in absolute value (Brazilian Reais). For example, 94.2% of the households claim to have purchased food in the previous month, spending on average R$323.90. We observe that 27.1% of households bought soda, with average spending of R$22.50, and 22% had some expenses with tobacco products, with an average of R$42.02.

**Table 2:** Average expenditure by household.

**Product**	**Any expenditure (%)**	**Expenditure (R**$**)**
Total food	94.2	323.90
Total food at home	91.0	263.16
Soda	27.1	22.50
Cookies	36.2	19.25
Packaged food	9.0	37.21
Food away from home (unhealthy)	38.2	43.22
Food away from home (total)	59.4	104.58
Alcohol	13.4	67.18
Smoking	22.0	42.02

We perform a preliminary analysis by computing the correlation among these variables to check if they are highly correlated. A large correlation coefficient may indicate that the variables in question are part of a broader dimension, which should be identified by a factor analysis. Since the largest correlation coefficient is 0.25, the performance of a factor analysis is not necessary.

### 3.2 Methodology

The main objective of our analysis is to quantify if recipients of the cash transfer program increase their spending on ultra-processed foods with low nutritional value. In addition, we also investigate if the consumption of highly addictive goods such as alcohol and tobacco increases relative to similar households that do not receive monetary assistance. Different from the existing studies of *Bolsa Família*, we rely on the PSM to address these questions. This method provides an estimate of the effect of a treatment conditional on covariates that may help predict receiving the treatment [24]. As a result, the PSM estimates experience the effects of less selection bias and are recommended for observational studies and program evaluation [25].

The first step of the PSM is obtaining the propensity score, which is the probability of receiving the treatment, conditional on a set of covariates. By conditioning the probability of treatment on a set of covariates, we aim to make the treatment independent from the covariates. In our study, these covariates are variables that can predict being a recipient of the cash transfer. Thus, the household variables we take into consideration are the family size, number of children, household income, geographic region, whether the household is located in an urban area, whether the household is below the poverty or the extreme poverty line, whether someone in the household is pregnant or breastfeeding. In addition, we control for the *household reference person* variables of age, gender, race, and schooling level.

In the second step, we use the calculated propensity score to perform a nearest-neighbor matching operation, where each treated unit is matched with an untreated unit. In our study, we match a household that participates in *Bolsa Família* with another household that does not receive the funds from the program but shares roughly the same propensity score. This is possible because some of the eligible families do not participate in the program due to the limited number of quotas allocated to their municipality by the federal government.

To measure the effects of unhealthy expenditure, we evaluate their sensitivity by computing the extensive and intensive margins. In our study, an extensive margin refers to the difference in the percentage of a product purchased between participants and nonparticipants of *Bolsa Família*.

An intensive margin considers the monetary difference spent on a specific product between participants and nonparticipants. Since the expenditure data is right-skewed, we apply a log-transformation to it to obtain an empirical distribution closer to a normal distribution. Notice that by using the log-transformation, we are restricted to comparing the percentage difference in expenditure only among households who have purchased the product in question. Following the log-transformation, the skewness of all variables lies between the ideal range of −0.5 and 0.5 for the distribution to be considered approximately symmetric, except for the variables *total food expense* (-0.87) and *household food expense* (-0.69).

#### 3.2.1 Computing the propensity score with machine learning methods

While logistic and probit regressions are the predominant methods for propensity score estimation [8], some recent studies have shown that machine learning techniques can produce estimates with lower sensitivity and accuracy. The reason is that these techniques capture nonlinear relationships among the investigated variables, resulting in better estimates of the propensity score [26].

As mentioned in Section 3.2, the first step of the PSM is obtaining the propensity score. In our study, we obtain this estimate using four different machine learning binary classification models and check which algorithm produces the propensity score estimation with the lowest sensitivity and misclassification. The methods we evaluate are random forests, gradient boosting, SVM, and neural networks.

While the study of Westreich et al. [27] argues that the predictive accuracy by some of these methods improves relative to the logistic regression, we are unaware of a study conducting a horse race among these methods in the context of the observational data of *Bolsa Família*. A few related studies compare multiple machine learning models using simulated data [28], [29].

After estimating a propensity score using each of the proposed methods, we compute their sensitivity, accuracy, and misclassification rate, defined as, respectively,

(1) $$\begin{array}{c} \mathrm{Sensitivity}=\frac{\mathrm{TP}}{P},\;\;\;\\ \mathrm{Accuracy}=\frac{\mathrm{TP}+\mathrm{TN}}{P+N}, \end{array}$$

MisclassificationRate=1-Accuracy,

where the true positive TP is the number of households correctly classified as *Bolsa Família* participants, the true negative TN is the number of households correctly classified as non-*Bolsa Família* participants, the condition positive P is the total number of *Bolsa Família* participants, and the condition negative N is the total number of non-*Bolsa Família* participants.

It is important to highlight that the sensitivity measure in (1) captures the fraction of correctly classified participants in the universe of all participants of *Bolsa Família*. The reason why this measure is critical to us is that there are eligible households in our sample who would have been classified by the machine learning algorithms as recipients of the *Bolsa Família* funds but were unable to participate in the program due to the limited available quotas. For this reason, the sensitivity measure reflects more accurately the ability of the machine learning methods to correctly classify the participants of *Bolsa Família* than the misclassification rate or accuracy, two measures that are widely adopted in the machine learning literature [30], [31]. We use 70% of the data set for training, 10% for validation, and the remaining 20% for testing to address the issue of overfitting.

Table 3 shows the sensitivity, accuracy, and misclassification rate for each of the five models. As illustrated, gradient boosting has the highest sensitivity and accuracy (93.5% and 76.5%, respectively) among all models, followed by random forests (83.3% and 76.3%, respectively) and neural networks (77.6% and 75.4%, respectively). We highlight that despite the gradient boosting method and random forest having roughly the same accuracy, they differ more than 10% in their sensitivity, which indicates that the fact that gradient boosting is considerably more accurate when restricting the sample space to those who participate in *Bolsa Família*. Another surprising result is that the logistic regression–the most used method in the estimation of propensity scores–displays the lowest sensitivity and accuracy (76.5% and 74.2%) among all models tested.

**Table 3:** Model Comparison.

**Model**	**Sensitivity(%)**	**Accuracy(%)**	**Misclassification(%)**
Gradient Boosting	93.5	76.5	23.5
Random Forests	83.3	76.3	23.7
Neural Networks	77.6	75.4	24.6
SVM	76.6	74.2	25.8
Logistic Regression	76.5	74.2	25.8

Given the results presented in Table 3, we use the gradient boosting method in the first stage of the PSM to obtain the propensity score, which is essentially the probability of being a *Bolsa Família* participant conditional on a given set of variables we controlled for (i.e., the covariates). Once the probabilities are obtained, we proceed to the second stage of the PSM, where we match observations with similar propensity scores.

**Fig. 1:**
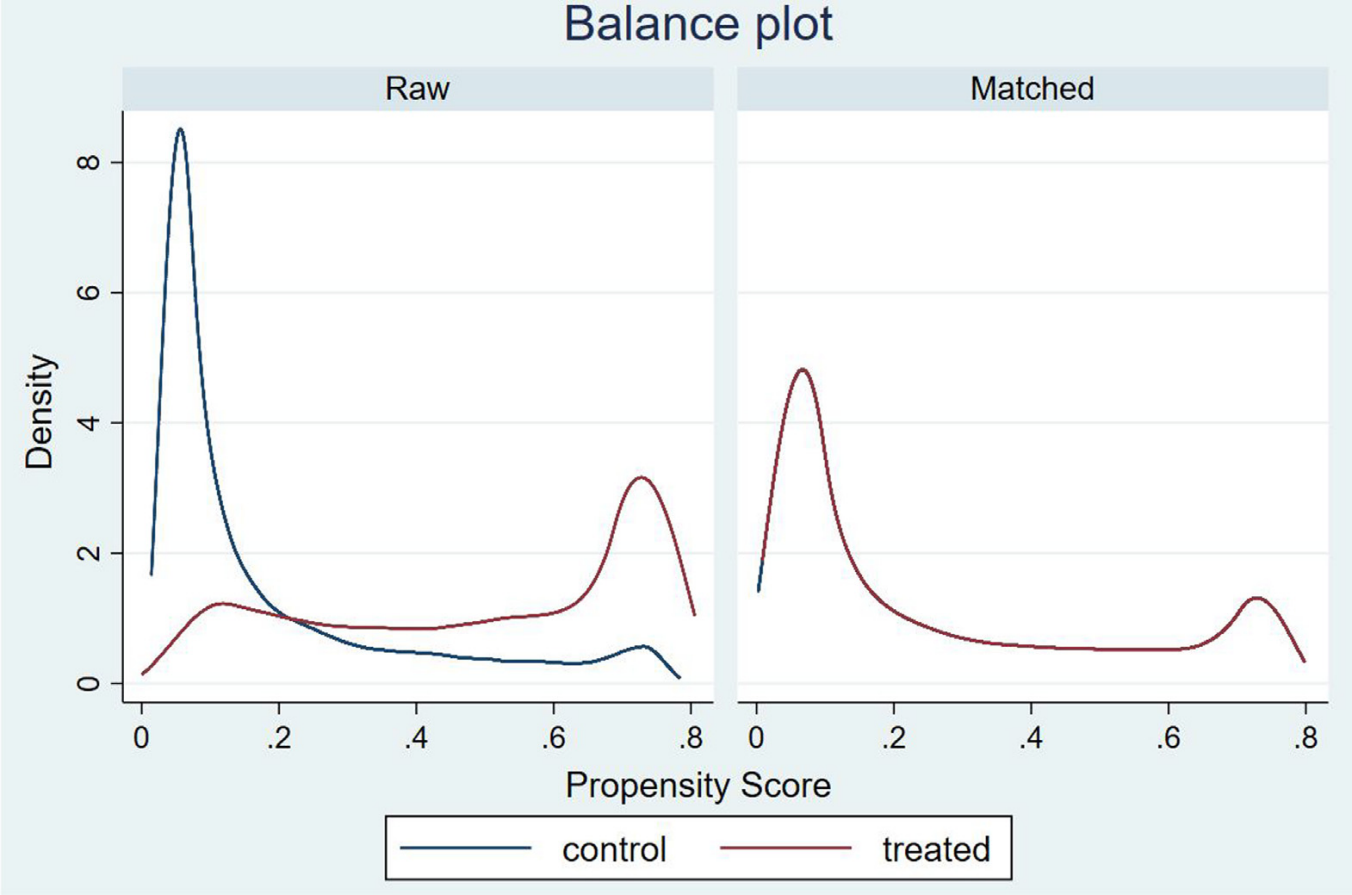
*Propensity scores density plots.* The graphs show the propensity scores’ densities of the full sample (i.e., raw) on the left-hand side and the matched sample on the right-hand side of the control (blue) and treated (red) groups.

After estimating the propensity scores and before proceeding to the results, the common support should be verified. Common support is the overlap in the range of propensity scores across *Bolsa Família* participants and nonparticipants and it is visually assessed in the graph of propensity scores across these groups [32]. Fig. 1 presents the density plots of the propensity scores of the cash-transfer recipients (treated group, red line) and nonrecipients (control group, blue line). The left panel shows the densities for the controlled and treated groups using the full sample data (labeled as raw). As illustrated, most of the households that do not participate in the program (control group) are assigned a probability of 10% of receiving *Bolsa Família*, indicating that nonrecipients are correctly predicted not to partake in the program. Similarly, most of the households that participate in the program (treated group) receive a probability of participating of 75%. Before proceeding to match these groups, they should be balanced, i.e., the distribution of propensity scores should be similar across both groups. The panel on the right displays the density of the matched sample, and we can observe a great overlap of propensity scores across *Bolsa Família* participants and nonparticipants.

**Fig. 2:**
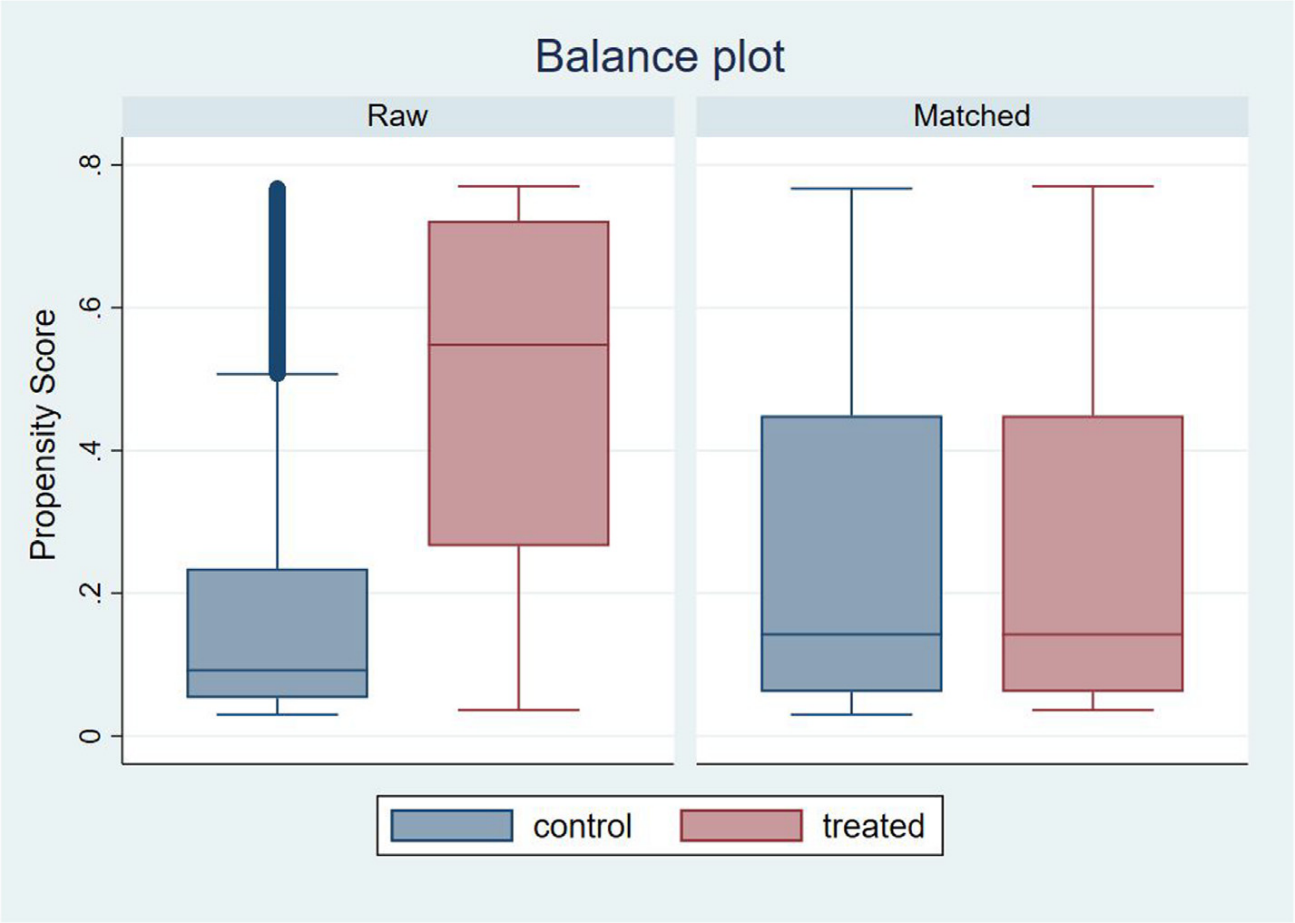
*Propensity scores box plots.* The graphs show the box plots for the full sample (i.e., raw) on the left-hand side and the matched sample on the right-hand side of the control (blue) and treated (red) groups.

To check the balance in matched samples, we provide in Fig. 2 the box plots for the propensity scores, which graphically evaluate the quality of the match. The box plots for recipients are marked in red and nonrecipients in blue. The panel on the left-hand side shows the balance plot of the propensity scores for the full sample data (labeled as raw). The great disparity between these groups indicates that nonrecipients (control group) are predicted with a low probability of participating in the program, while recipients (treated group) are predicted with a higher probability of participating in the program. The panel on the right shows the balance plot for the propensity score for the matched group. Notice that the matched sample displays considerably more balanced box plots between recipients and nonrecipients of the cash-transfer program.

### 3.3 Results

We estimate two PSM models to capture the effects of the outcomes at the extensive and intensive margins. The extensive margin captures the change in consumption, measured as the percentage differences in household purchases between participants and nonparticipants of *Bolsa Família*. More specifically, the measure reflects how much more (or less) a participant household spends buying an unhealthy product in comparison to a nonparticipant household. To estimate the extensive margin, we use a dummy variable that assumes value 1 when the household has purchased the product in the past month.

The *intensive margin* captures the percentage increase (or decrease), measured in Brazilian Reais, a participant household spends buying an unhealthy product relative to a nonparticipant household, given that the household has purchased the unhealthy item. We estimate the effects on intensive margins using the log-transformation of the monetary expenditure to reduce the skewness of the distribution. Table 4 presents the average treatment effect (ATE hereafter), the standard error (SE hereafter) of the extensive margin model (first column), and the intensive margin model (second column). We observe that the total food consumption increases by 2 percentage points (pp hereafter) while the total food at home increases by 3.3 pp. The consumption of cookies, unhealthy food away from home, and total food away from home also experience an increase of 4.8 pp, 7.5 pp, and 5.7 pp, respectively. Notice that the consumption of unhealthy food away from home experiences the largest increase in pp among all products we investigate. Surprisingly, soda consumption is the only item that experiences a statistically significant decline in consumption of 3.3 pp, while the decline in the consumption of packaged food is not significant. Intensive margin results show that, among those who purchased food in the last month, *Bolsa Família* participants increase their total spending on food by 14.5% and total food purchased for consumption at home by 10.4%, in line with the findings in the literature [16], [33]. Interestingly, according to the intensive margin model, there is no significant difference in monetary expenses on the products analyzed between participants and nonparticipants. The last two rows of Table 4 reveal that the increase in consumption of alcohol and tobacco products is not significant in the models investigated, indicating that recipients of the cash transfer program are not necessarily engaging in risky consumption behaviors more than nonparticipants.

**Table 4:** Gradient boosting: Marginal effects on food, alcohol, and smoking spending.

**Model**	**Extensive Margin**		**Intensive Margin**	
	ATE	SE	ATE	SE
Total food	$0.020^{***}$	0.004	$0.145^{***}$	0.038
Total food at home	$0.033^{***}$	0.006	$0.104^{***}$	0.039
Soda	$-0.033^{***}$	0.012	−0.058	0.042
Cookies	$0.048^{**}$	0.021	0.041	0.029
Packaged food	−0.010	0.012	−0.126	0.090
Food away from home (unhealthy)	$0.075^{***}$	0.022	−0.016	0.040
Food away from home (total)	$0.057^{***}$	0.022	−0.034	0.042
Alcohol	0.003	0.012	0.019	0.122
Tobacco	0.005	0.014	−0.094	0.054

*Note*: The table displays the ATE and SE for the extensive and intensive margin models. Each ATE cell represents a separate regression. *** denotes that the estimate is statistically significant at 1%, and ** denotes that the estimate is statistically significant at 5%.

Lastly, as a robustness check, we compare the marginal effects on food, alcohol, and smoking spending of the selected model, gradient boosting, with the standard logistic regression model. Table 5 presents the results. Overall, the average treatment effects of the extensive and intensive margins are consistent (in value and significance) with a few exceptions. Using the logistic regression model, soda consumption is not significant, but consumption and expenditure on tobacco are. Tobacco consumption increases by 2.2 pp while tobacco spending decreases by 15%.

**Table 5:** Logistic regression: Marginal effects on food, alcohol, and smoking spending.

**Model**	**Extensive Margin**		**Intensive Margin**	
	ATE	SE	ATE	SE
Total food	$0.022^{***}$	0.004	$0.134^{***}$	0.017
Total food at home	$0.033^{***}$	0.005	$0.103^{***}$	0.017
Soda	0.009	0.008	−0.026	0.023
Cookies	$0.043^{***}$	0.008	−0.011	0.021
Packaged food	0.008	0.006	−0.107	0.068
Food away from home (unhealthy)	$0.068^{***}$	0.008	$-0.051^{**}$	0.027
Food away from home (total)	$0.074^{***}$	0.008	−0.039	0.028
Alcohol	0.001	0.006	0.010	0.048
Tobacco	$0.022^{***}$	0.007	$-0.150^{***}$	0.034

*Note*: The table displays the ATE and SE for the extensive and intensive margin models. Each ATE cell represents a separate regression. *** denotes that the estimate is statistically significant at 1%, and ** denotes that the estimate is statistically significant at 5%.

## 4 Conclusion

Our study shows that when compared to a similar household that does not participate in the Brazilian CCT program, a *Bolsa Família* participant household spends more funds purchasing food, but the differences in unhealthy food product expenses are not significant among families that already buy those. We also document that the probability of purchasing cookies and food away from home increases, according to the extensive margin model.

We find that there are no significant differences in the probability of purchasing alcohol and tobacco products. In addition, we find that there are no significant differences in the number of funds spent on these products as well, indicating that recipients of the CCT program do not engage in more risky consumption behaviors than nonrecipients. We show that the only unhealthy product that experiences a decline in the probability of being purchased is soda.

Overall, our study finds that the participants in *Bolsa Família* use the cash transfer to purchase more food, but they do not necessarily spend the funds on unhealthy products that could potentially worsen their diets and lead to long-term health complications. We argue that our results are particularly robust due to our proposed methodology of estimating the propensity score through a machine learning model horse race. We propose selecting the machine learning model with the highest sensitivity and the least misclassification. While machine learning models are generally criticized for the nontrivial interpretability of their output, estimating a propensity score for matching purposes does not require interpretation, and this criticism does not apply to our case. We find that gradient boosting outperforms all the competing models and that the most widely used method in the literature–logistic regression–has the lowest sensitivity and accuracy among all the tested models.

## Declaration of Competing Interest

The authors declare that they have no known competing financial interests or personal relationships that could have appeared to influence the work reported in this paper.
