# Supplementary material for: The impact of cash transfer participation on unhealthy consumption in Brazil
Source: Health Policy Open. 2022 Dec 6;4:100087. doi: 10.1016/j.hpopen.2022.100087 (PMC10297731; doi:10.1016/j.hpopen.2022.100087)
Supplement: Supplementary file 1 [file mmc1.pdf]

Supplementary Material of The Impact of Cash Transfer Participation on Unhealthy Consumption in Brazil

November 30, 2021

Additional Tables and Figures

Figure 1 presents the density plots of the propensity scores and the overlapping of the propensity scores of the cash transfer recipients (treated group) and nonrecipients (control group). The left panel shows the densities for the controlled and treated groups using the full sample data (labeled as raw). As illustrated, the density of low propensity scores among nonrecipients is very large, which indicates that households that do not participate in the program, mostly because they are not eligible, are correctly attributed with a low probability of being a participant. Similarly, most of the households that participate in the program receive a propensity score of about 70% to 75%. The panel on the right displays the density of the matched sample or the common support condition, which is the overlap range of propensity scores across *Bolsa Família* participants and nonparticipants. The overlap indicates that the matched groups share similar covariate values as if they were drawn from a randomized experiment (see [1]).

A critical step of the PSM is to ensure that the matched samples are balanced, i.e., the distribution of propensity scores should be similar across both groups. To illustrate the

Table 1: Machine Learning Methods Comparison.

Method	Advantages	Limitations
**Logistic Regression**	Easy to understand and interpret, accessible	Does not handle missing data, assumes linear relationships, unreliable fit tests
**Random Forest**	Nonparametric, handles missing data well, incorporates interactions, strong predictive power, robust to outliers	Interpretability, requires computational resources
**Gradient Boosting**	Nonparametric, less susceptible to overfitting, can handle high-dimensional data, handles any variable type and missing data, robust to outliers	Fairly complex, requires intense computational resources, interpretability
**SVM**	Nonparametric, high-dimensional data, incorporates interactions, robust to overfitting	Does not perform well with noisy data, not suitable for large datasets, trade-off accuracy vs. generalization, kernel selection
**Neural Networks**	Nonparametric, can handle high-dimensional and missing data	Requires inputs for hidden layer and training procedures, can overfit

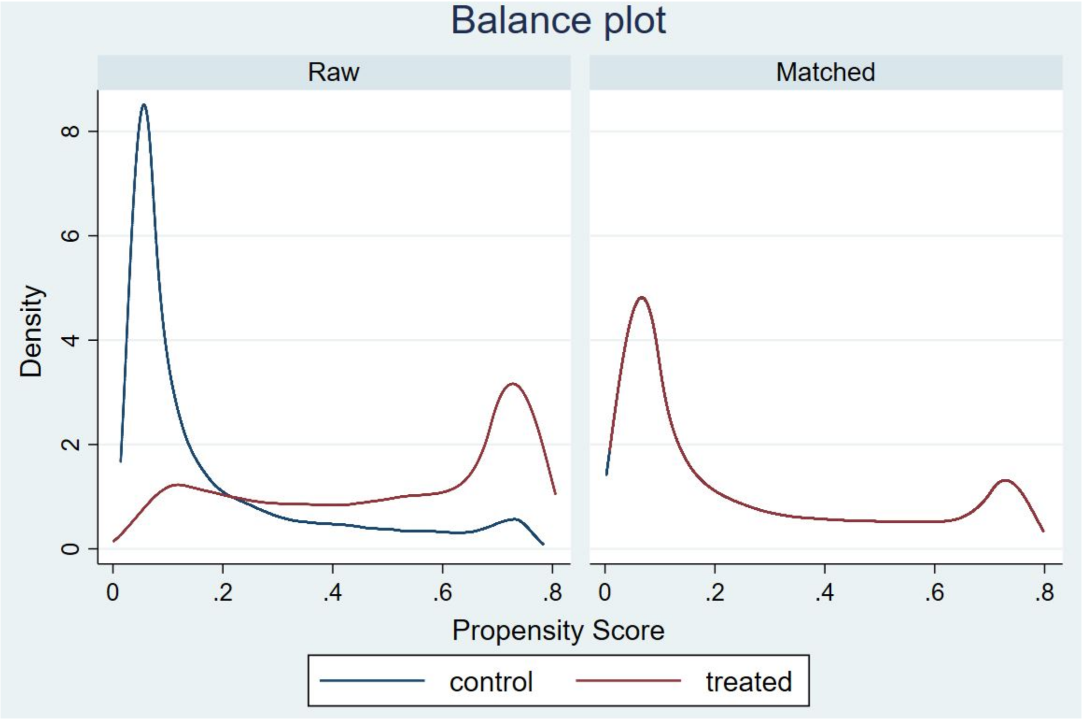

Figure 1: *Propensity scores density plots.* The graphs show the propensity scores' densities of the full sample (i.e., raw) on the left-hand side and the matched sample on the right-hand side of the control (blue) and treated (red) groups.

quality of the match and check the balance in matched samples, we provide the box plots for the propensity scores in Figure 2. The panel on the left shows the balance plot of the propensity scores for the full sample data (i.e., raw). The large disparity between these two groups indicates that our classification model attributes significantly different scores to predicted participants and nonparticipants of the *Bolsa Família* program. The panel on the right shows the balance plot for the propensity score of the matched group. As illustrated, the matched sample displays considerably more balanced box plots between recipients and nonrecipients of the cash transfer program.

Lastly, to verify the matching quality, we check the balance of the covariates via the standard percentage bias (see [2]). According to [3], we can say that there is a covariate balance in the matched sample if the percent reduction in bias is less than 20%. Figure 3 shows the standardized percentage differences across covariates. The circles represent the standardized percentage bias of the full sample, and the crosses represent the standardized

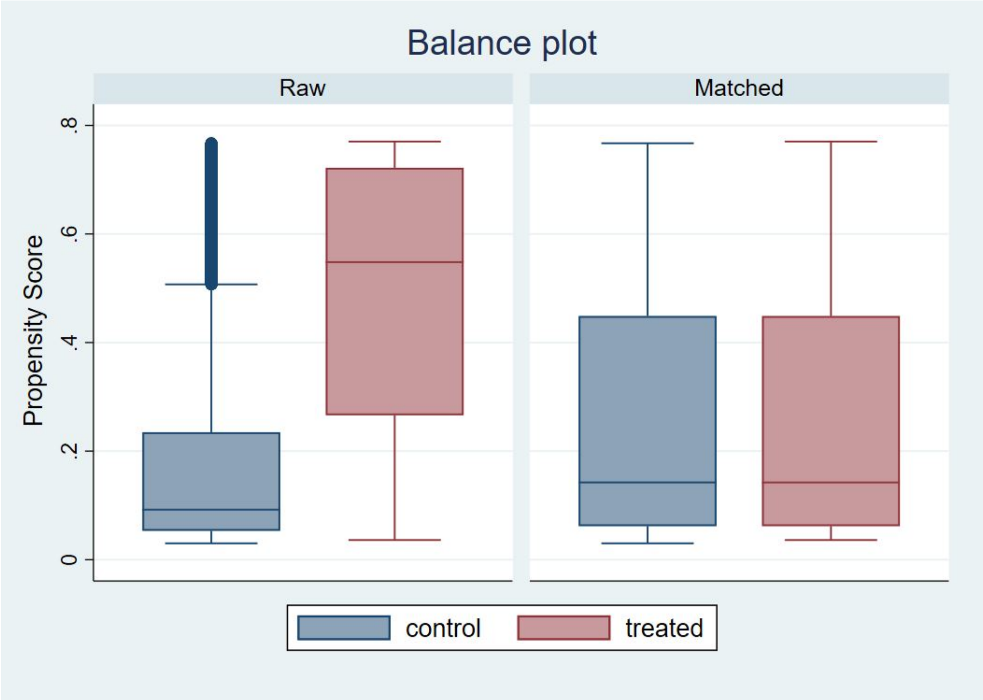

Figure 2: *Propensity scores box plots.* The graphs show the box plots for the full sample (i.e., raw) on the left-hand side and the matched sample on the right-hand side of the control (blue) and treated (red) groups.

percentage bias of the matched sample. As illustrated, the full sample percentage bias ranges from -103% to 87%, which are much higher than the maximum threshold of 20% for standardized differences of bias across covariates, as suggested by [3]. In contrast, notice that the percentage bias for the matched data sample ranges from -7% to 4%. Overall, the graphs indicate that the propensity score distribution of the matched sample data is balanced, and we can proceed to analyze the effect estimation.

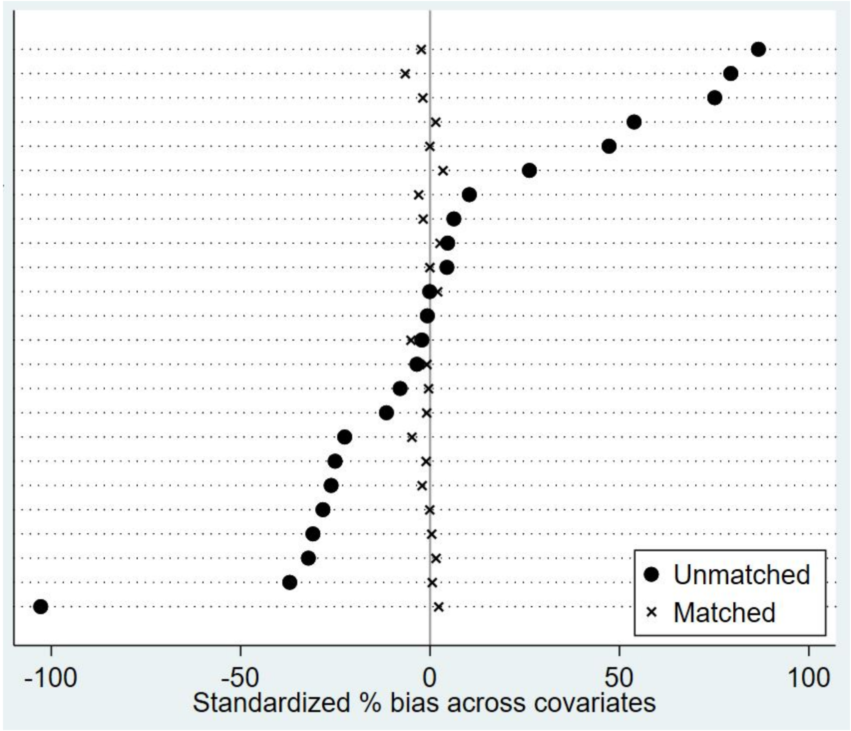

Figure 3: Standardized percentage bias across covariates.

References

[1] L. Grilli and C. Rampichini, "Propensity scores for the estimation of average treatment effects in observational studies," *Training Sessions on Causal Inference, Bristol*, pp. 28–29, 2011.

[2] M. Caliendo and S. Kopeinig, "Some practical guidance for the implementation of propensity score matching," *Journal of Economic Surveys*, vol. 22, no. 1, pp. 31–72, 2008.

[3] P. R. Rosenbaum and D. B. Rubin, "Constructing a control group using multivariate matched sampling methods that incorporate the propensity score," *The American Statistician*, vol. 39, no. 1, pp. 33–38, 1985.
